# Lancing Gums

**Published:** 1861-02

**Authors:** Julius Dienelt


Lancing Gums.—By *Julius Dienelt.*—Having read an article on lancing gums, by Dr. J. D. White, in a previous number of the Dental Cosmos, in which he advocates lancing the gums before extracting teeth, and knowing how indispensable it is to do so, we would give the following case in illustration of the aforesaid. It happened to us but a few weeks ago:

The patient being a youth about sixteen years of age. He applied to us for the extraction of the first right inferior molar. As the tooth in question appeared to be set very firmly, we lanced the gums well all around, except between it and the second bicuspid, where we could not get with our lancet; the forceps were applied, and the tooth drawn quite readily. Upon examining the tooth, however, we were surprised to see the bicuspid firmly united to it, by a flap of hard granulated gum; both teeth had thus been drawn simultaneously. So firmly was the gum united to both teeth, that it required considerable force to cut it loose from them.

The tooth thus accidentally dislodged proved to be perfectly sound, so that we thought it best to slip it back into its sockit, requesting the patient to inform us in a short time how the tooth was getting on. Up to this time the tooth is doing well, and has become quite firm again. We have made up our mind, since this occurrence, which at the time we regretted very much, to be more careful than ever to lance the gums well before extracting teeth.—*Dental Cosmos.*

